# Variable Temporal Cerebral Blood Flow Response to Acetazolamide in Moyamoya Patients Measured Using Arterial Spin Labeling

**DOI:** 10.3389/fneur.2021.615017

**Published:** 2021-06-08

**Authors:** Markus Fahlström, Johan Wikström, Ljubisa Borota, Per Enblad, Anders Lewén

**Affiliations:** ^1^Department of Surgical Sciences, Radiology, Uppsala University, Uppsala, Sweden; ^2^Department of Neuroscience, Neurosurgery, Uppsala University, Uppsala, Sweden

**Keywords:** moyamoya disease, magnetic resonance imaging, cerebral blood flow, perfusion imaging, cerebrovascular reserve

## Abstract

Cerebrovascular reserve capacity (CVR), an important predictor of ischaemic events and a prognostic factor for patients with moyamoya disease (MMD), can be assessed by measuring cerebral blood flow (CBF) before and after administration of acetazolamide (ACZ). Often, a single CBF measurement is performed between 5 and 20 min after ACZ injection. Assessment of the temporal response of the vasodilation secondary to ACZ administration using several repeated CBF measurements has not been studied extensively. Furthermore, the high standard deviations of the group-averaged CVRs reported in the current literature indicate a patient-specific dispersion of CVR values over a wide range. This study aimed to assess the temporal response of the CBF and derived CVR during ACZ challenge using arterial spin labeling in patients with MMD. Eleven patients with MMD were included before or after revascularisation surgery. CBF maps were acquired using pseudo-continuous arterial spin labeling before and 5, 15, and 25 min after an intravenous ACZ injection. A vascular territory template was spatially normalized to patient-specific space, including the bilateral anterior, middle, and posterior cerebral arteries. CBF increased significantly post-ACZ injection in all vascular territories and at all time points. Group-averaged CBF and CVR values remained constant throughout the ACZ challenge in most patients. The maximum increase in CBF occurred most frequently at 5 min post-ACZ injection. However, peaks at 15 or 25 min were also present in some patients. In 68% of the affected vascular territories, the maximum increase in CBF did not occur at 15 min. In individual cases, the difference in CVR between different time points was between 1 and 30% points (mean difference 8% points). In conclusion, there is a substantial variation in CVR between different time points after the ACZ challenge in patients with MMD. Thus, there is a risk that the use of a single post-ACZ measurement time point overestimates disease progression, which could have wide implications for decision-making regarding revascularisation surgery and the interpretation of the outcome thereof. Further studies with larger sample sizes using multiple CBF measurements post-ACZ injection in patients with MMD are encouraged.

## Introduction

Moyamoya disease (MMD) is a cerebrovascular disorder characterized by progressive steno-occlusion of the terminal part of the internal carotid arteries (ICAs), which extends to the middle cerebral arteries (MCAs) and anterior cerebral arteries (ACAs). Cerebral perfusion pressure (CPP) may drop distal to the steno-occlusive lesion, resulting in impaired cerebral blood flow (CBF), which may lead to ischaemic stroke. Fragile collaterals (moyamoya vessels) are recruited at the base of the brain to maintain CBF and may cause life-threatening cerebral hemorrhage if they rupture (1). Moreover, reductions in CPP will trigger a haemodynamic and metabolic response to maintain adequate CBF and preserve the cerebral metabolic rate of oxygen (CMRO_2_) in patients with MMD (2). Cerebral autoregulation will dilate resistance arteries to maintain adequate CBF, and increases in oxygen extraction fraction will contribute sufficient oxygenation to maintain normal CMRO_2_. Further CPP reduction will result in insufficient delivery of oxygen to meet metabolic demands; CMRO_2_ will then decrease, and ischaemia may follow (2–4). Cerebrovascular reserve (CVR), which reflects the degree of vasodilatory autoregulation, is defined as the maximum percentage increase in CBF after a vasodilatory challenge (5). A commonly used vasodilatory agent is acetazolamide (ACZ), which, when given intravenously, will dilate resistant arteries (4, 5). Impaired CVR is associated with an increased risk of ischaemic events and transient ischaemic attacks and is also considered an indication for revascularisation surgery (6). In a recent meta-analysis outlining the association between CVR impairment and stroke risk in patients with carotid stenosis and occlusions, the non-standard definition of impaired and normal CVR and variability in post-ACZ measurement timing were highlighted as limitations (6). The temporal response to ACZ has not been studied extensively, and the maximum increase in CBF is reported to occur from 10 to 25 min after injection (7–11). Moreover, previous studies often report large standard deviations (SDs) of CVR in groups of patients with MMD and other steno-occlusive diseases (3, 9, 12–22). This indicates that individual CVR values in affected vascular territories are dispersed over a wide range, which may be explained by variance in temporal CBF response after ACZ administration. We therefore hypothesized that if only a single post-ACZ injection CBF measurement is used, the maximum increase in CBF may be missed and CVR underestimated. Thus, the aim of this study was to assess the individual temporal response of CBF after ACZ challenge in MMD patients using arterial spin labeling (ASL) magnetic resonance imaging (MRI).

## Methods

### Patients

Eleven patients with confirmed bilateral or unilateral MMD and an MRI examination before and/or after indirect revascularisation surgery were retrospectively included in this study. The MMD patients were graded by an experienced neurointerventionist using the Suzuki Score system (23) based on digital subtraction or magnetic resonance angiography (MRA). Affected ACAs and MCAs were included as affected vascular territories, posterior cerebral arteries (PCAs) were included as an unaffected vascular territory, and contralateral ACAs and MCAs in patients with unilateral MMD were included as unaffected contralateral vascular territories. Although both contralateral ACAs and MCAs and PCAs are considered to be unaffected, they are included separately due to transit time differences between the anterior and posterior circulation (24). For a detailed description of patient characteristics and included vascular territories, see Table 1. All patients received an intravenous ACZ injection, 1 g being given to adults and 10 mg/kg to children. This retrospective study was done in accordance with the declaration of Helsinki and was approved by the Swedish Ethical Review Authority, and all included patients or legal guardian signed an informed consent.

**Table 1:** Patient characteristics.

**#**	Age^*^/Sex	**Symptoms**	**Etiology**	**Side**	**Suzuki grade**	**Affected regions**	**Unaffected regions**	**Surgery**	**MRI**
1	14/M	TIA R side	MMD	L	L ICA: II	L ACA + MCA	Bilat PCA	L MBH	|Post|Post|Post|
2	8/F	TIA R side + speech	MMD	Bilat	R/L ICA: II-III	Bilat ACA + MCA	Bilat PCA	R/L MBH	|Post|Post^‡^|Post|
3	52/F	TIA	MMD	Bilat	R/L ICA: IV	Bilat ACA + MCA	Bilat PCA	R/L MBH	|Post|Post|Post|
4	25/F	-	NF1	R	R ICA: IV-V	R ACA + MCA	Bilat PCA	R MBH	|Pre|Post|Post|
5	27/F	Paraesthesia Headache	MMD	Bilat	R/L ICA: III	R ACA Bilat MCA	Bilat PCA	R MBH	|Pre|Post|
6	26/F	Paraesthesia Headache	MMD	Bilat	R ICA: IV L ICA: III	Bilat ACA + MCA	Bilat PCA	L MBH	|Pre|Pre|Post|
7	33/F	Facial pain	Atypical MMD	R	R ICA: III	Right ACA + MCA	Bilat PCA		|Pre|Pre|
8	51/F	SAH	MMD	Bilat	R/L ICA: III	Bilat ACA + MCA	Bilat PCA		|Pre|
9	18/F	Infarct	MMD	Bilat	R/L ICA: IV	Bilat ACA + MCA	Bilat PCA		|Pre|
10	22/F	-	MMD	Bilat	R/L ICA: IV^†^	Bilat ACA + MCA	Bilat PCA	Bypass	|Post|Post|
11	14/F	Paraesthesia Headache	MMD	Bilat	R/L ICA: III^†^	Bilat ACA + MCA	Bilat PCA		|Pre|

* *Age at first examination*.

† *Suzuki grade assessment by magnetic resonance angiography*.

‡ *Bilateral PCA excluded due to insufficient imaging volume coverage*.

*Bilat, bilateral; F, female; ICA, internal carotid artery; L, left; M, male; MMD, moyamoya disease; MRI, magnetic resonance imaging; NF, neurofibromatosis 1; PCA, posterior cerebral artery; Pre, preoperative examination; Post, postoperative examination; R, right; SAH, subarachnoid hemorrhage; TIA, transient ischaemic attack*.

### Magnetic Resonance Imaging

All examinations were performed on a Philips Achieva, 3.0 T (Philips Healthcare, Best, the Netherlands) using a 32-channel head coil. A three-dimensional (3D) pseudo-continuous ASL (pCASL) with background-suppressed gradient spin-echo read-out using a post-label delay of 2,500 ms and label duration of 1,800 ms was acquired. Acquisition duration was 5 min and 31 s, with a repetition time of 4,735 ms and echo time of 10,7 ms, spatial resolution was 3 × 3 × 6 mm^3^, and the number of slices was 14. The acquisition included two background suppression pulses, and no flow-crushing gradients were applied. The labeling plane was placed perpendicular to the brain feeding arteries with the aid of a phase-contrast MRA survey. The 3D pCASL acquisition was performed before (baseline) and repeated 5, 15, and 25 min after ACZ injection. CBF maps were automatically calculated by the scanner according to the model recommended by Alsop et al. (25). Visual quality control was performed on all control/label pairs and calculated CBF maps. No exclusions were made due to head motion, and no venous hyperintensities were found in major venous structures and cortical veins. High signal intravascular arterial transit time artifacts were generally found; however, we have previously shown that these have a negligible effect upon CBF quantification using the same method as described here (26). Structural 3D T2-weighted fluid-attenuated inversion recovery (FLAIR) and 3D contrast-enhanced T1-weighted (CE-T1WI) images were acquired with spatial resolution 0.625 × 0.625 × 0.560 and 0.938 × 0.938 × 1 mm^3^ for tissue segmentation and registration purposes, respectively. Furthermore, a 3D time-of-flight MRA (spatial resolution 0.23 × 0.23 × 0.5 mm^3^) was included and used for Suzuki grade assessment if a digital subtraction angiography was missing.

### Image Post-processing

All individual calculated CBF maps and FLAIR images were registered to each patient's corresponding CE-T1WI image and resliced to its spatial resolution using trilinear interpolation. GM probability maps were segmented using a multimodal approach based on CE-T1WI and the registered FLAIR images (27). GM maps were defined with a partial volume fraction above 75%. The inverse deformation field, defining the transformation from Montreal Neurological Institute template space to patient-specific space, was derived based on each patient's CE-T1WI image and applied to a standard vascular territory template (28), including bilateral ACAs, MCAs, and PCAs. Each patient-specific vascular territory was resliced to the spatial resolution of the CE-T1WI image using nearest neighbor interpolation and then masked with the corresponding GM map. CBF values were extracted from the CBF maps using the masked vascular territory regions; hence, correction for partial volume effects was performed using the GM threshold method at CE-T1WI spatial resolution (29). All processing steps, as described earlier, were performed using the SPM12 toolbox (Wellcome Trust Centre for Neuroimaging, London, UK) and to ensure quality that was inspected visually.

### Statistical Analysis

CVR values were calculated based on average CBF per vascular territory, using Equation (1) for all post-ACZ injection examinations acquired. Absolute differences between time points for both CBF (CBF_Diff_) and CVR (CVR_Diff_) were calculated using the later post-injection time point as minuend and an early time point as subtrahend for all possible combinations, i.e., 15–0 or 15–5 min (clarified by superscript $CBF_{Diff}^{15-0}$ or $CVR_{Diff}^{15-5}$ when applicable).

(1) $$\begin{array}{r} CVR=\frac{CBF_{post\text{-}ACZ}-CBF_{baseline}}{CBF_{baseline}}\times 100\% \end{array}$$

For descriptive analysis of CBF and CVR, CBF_Diff_, and CVR_Diff_ average and SD were calculated for all vascular territories. A repeated-measures one-way analysis of variance (ANOVA) was performed to compare CBF and CVR values, and the Tukey's test was used to correct for multiple comparisons (not performed on unaffected contralateral vascular territories due to a small number of data points for individual vascular territories).

The time point for maximum increase in CBF, i.e., the highest increase in CBF post-ACZ injection occurring at 5, 15, or 25 min, was recorded for each examination and vascular territory. The sum of all counted occurrences at a given time point was normalized to the total number of examinations for each vascular territory and presented in percentage. Furthermore, “missing peaks” were defined as maximum increase in CBF occurring at 5 or 25 min post-ACZ injection in affected vascular territories, reflecting potential underestimation of CVR when performed as a single measurement at 15 min post-ACZ injection. To evaluate the effects of overlooking the actual maximum increase in CBF, the mean difference and range in percentage points (pp) between the missing peak CVR and CVR at 15 min was calculated. Furthermore, a paired *t*-test was performed to test whether the difference was significant. Derived *p-*values are two-sided and presented as exact values or <0.01 if below 0.01, where *p* < 0.05 is considered statistically significant. GraphPad Prism 8 for Mac (GraphPad Software, La Jolla, CA, USA) was used for statistical analysis and graph design.

## Results

### Patients

**Figure 1:**
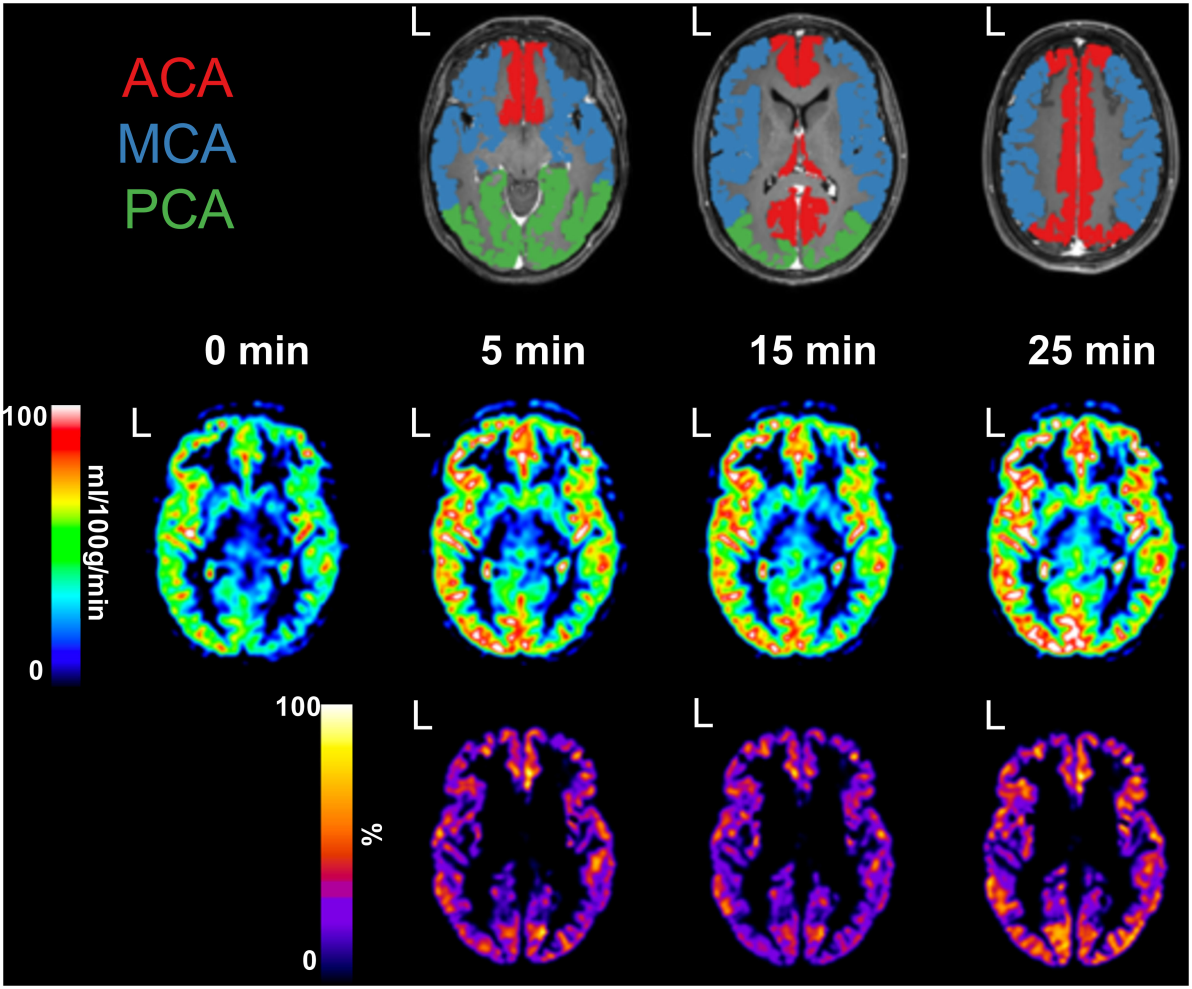
Preoperative examination of a female patient, aged 26 years, with confirmed bilateral moyamoya disease [Suzuki grade III left internal carotid artery (ICA) and grade IV right ICA]. An anatomical contrast-enhanced T1-weighed image shows defined vascular territories, including bilateral anterior cerebral artery (ACA, red), middle cerebral artery (MCA, blue), and posterior cerebral artery (PCA, green). L, denotes left side. Cerebral blood flow maps were acquired at baseline and 5, 15, and 25 min post-acetazolamide injection. Cerebrovascular reserve maps were calculated for each time point and presented masked with all vascular territories combined.

Twenty-four examinations (nine preoperative and 15 postoperative) were performed in 11 patients. For a detailed description of examinations and patient characteristics, see Table 1. Five patients were examined three times, three patients were examined twice, and three patients were examined once. Furthermore, three of the 11 patients had both pre- and postoperative examinations. Eight patients had bilateral disease, and three patients had unilateral disease (one left and two right hemispheres). A representative example of CBF maps at baseline and post-ACZ injection with corresponding CVR maps, together with an anatomical CE-T1WI image and derived vascular territories, is presented in Figure 1.

### Group Average

Average baseline CBF (SD) values were 38 (10) ml/100 g/min in affected, 32 (9) ml/100 g/min in unaffected, and 40 (9) ml/100 g/min in unaffected contralateral vascular territories. Average CBF (SD) values post-ACZ injection were similar in all vascular territories: affected 54 (17) at 5 min, 54 (17) at 15 min, and 54 (17) ml/100 g/min at 25 min post-ACZ injection, unaffected 49 (13) at 5 min, 47 (14) at 15 min, and 47 (14) ml/100 g/min at 25 min post-ACZ injection, and unaffected contralateral 60 (15) at 5 min, 59 (14) at 15 min, and 57 (13) ml/100 g/min at 25 min post-ACZ injection. CVRs (SD) were 40 (18) at 5 min, 39 (17) at 15 min, and 38 (15) % at 25 min post-ACZ injection averaged over all affected vascular territories, and corresponding values for unaffected and unaffected contralateral vascular territories were 52 (20) and 53 (24) at 5 min, 47 (20) and 49 (18) at 15 min, and 44 (15) and 45 (12) % at 25 min post-ACZ injection, respectively.

**Figure 2:**
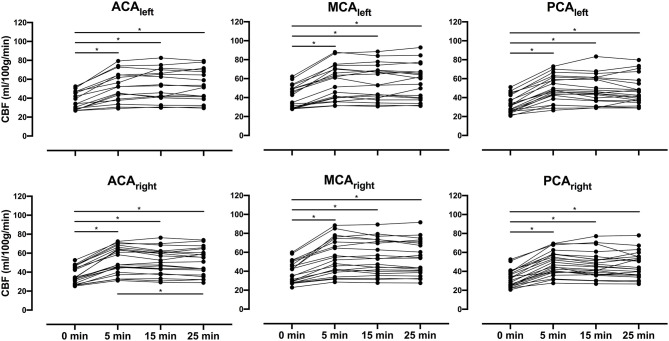
Presents individual cerebral blood flow (CBF) for all patients as spaghetti plots in bilateral anterior cerebral arteries (ACAs), middle cerebral arteries (MCAs), and posterior cerebral arteries (PCAs) for baseline (0 min) and all post-acetazolamide (ACZ) injection time points (5, 15, and 25 min). A repeated-measures one-way analysis of variance was performed between time points, and Tukey test was used to correct for multiple comparisons; significant difference (*p* < 0.05) is indicated with an asterisk (*).

**Figure 3:**
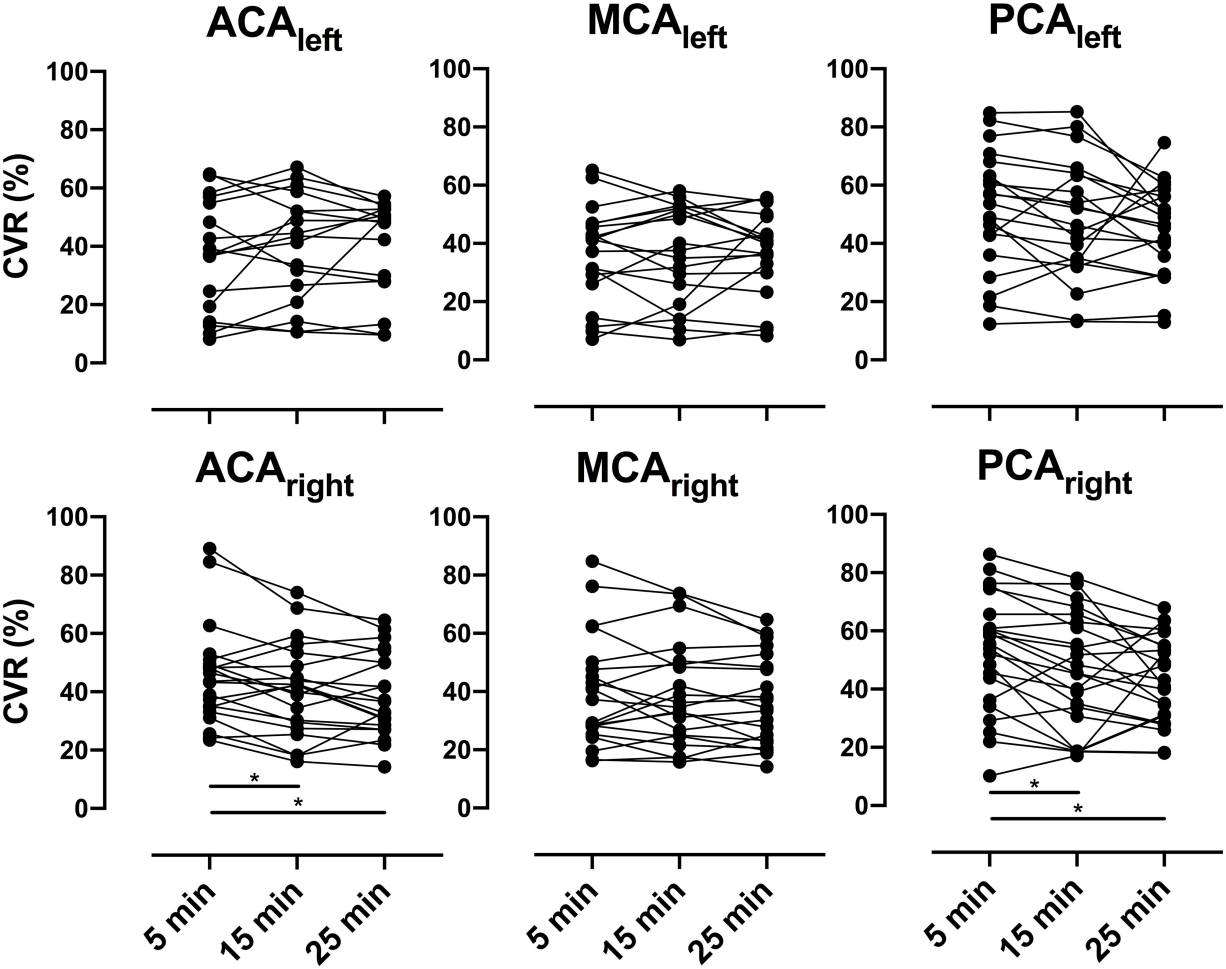
Presents individual cerebrovascular reserve (CVR) for all patients as spaghetti plots in bilateral anterior cerebral arteries (ACAs), middle cerebral arteries (MCAs), and posterior cerebral arteries (PCAs) for all post-acetazolamide (ACZ) injection time points (5, 15, and 25 min). A repeated-measures one-way analysis of variance was performed between time points, and Tukey test was used to correct for multiple comparisons; significant difference (*p* < 0.05) is indicated with an asterisk (*).

Average CBF increased significantly (*p* < 0.01) at all post-ACZ injection time points measured compared with baseline for each individual vascular territory. No significant differences were found for CBF between post-ACZ injection time points, except for $CBF_{Diff}^{25-5}$ in ACA_right_ (*p* = 0.03). For CVR, significant differences were observed for $CVR_{Diff}^{15-5}$ and $CVR_{Diff}^{25-5}$ in ACA_right_ (*p* = 0.04 and *p* < 0.01, respectively) and PCA_right_ (*p* < 0.01 and *p* = 0.04, respectively). Data for each vascular territory and average data over affected, unaffected, and unaffected contralateral vascular territories are presented in Table 2. Graphs showing individual data points for CBF are presented in Figure 2 and for CVR in Figure 3.

**Table 2:** Average CBF and CVR with standard deviation for each individual vascular territory and all affected, unaffected, and unaffected contralateral vascular territories and all post-ACZ injection time points.

	**Affected**					**Unaffected**			**CL**
**Time after injection**	**ACA_**left**_**	**ACA_**right**_**	**MCA_**left**_**	**MCA_**right**_**	**All**	**PCA_**left**_**	**PCA_**right**_**	**All**	**All**
**CBF (ml/100 g/min)**									
0 min	38 (10)	36 (9)	41 (12)	39 (11)	38 (10)	33 (9)	32 (9)	32 (9)	40 (10)
5 min	52 (16)	53 (14)	56 (19)	55 (19)	54 (17)	49 (13)	49 (12)	49 (13)	60 (15)
15 min	53 (17)	51 (13)	56 (18)	54 (18)	54 (17)	48 (15)	47 (13)	47 (14)	59 (14)
25 min	53 (17)	51 (14)	57 (19)	54 (18)	54 (17)	47 (15)	46 (13)	47 (14)	57 (13)
**CVR (%)**									
5 min	37 (19)	45 (17)	36 (17)	40 (19)	40 (18)	52 (20)	53 (20)	52 (20)	53 (24)
15 min	40 (19)	41 (16)	36 (17)	39 (18)	39 (17)	48 (20)	46 (20)	47 (20)	49 (18)
25 min	40 (17)	39 (14)	37 (15)	37 (15)	38 (15)	45 (16)	44 (15)	44 (20)	45 (12)

*ACA, anterior cerebral artery; CBF, cerebral blood flow; CL, unaffected contralateral vascular territories; CVR, cerebrovascular reserve; MCA, middle cerebral artery; PCA, posterior cerebral artery*.

### Individual Analysis

In most patients, the maximum increase in CBF was seen 5 min after injection, but this maximum at 5 min was more common in unaffected (59%) and unaffected contralateral vascular territories (61%) than in affected vascular territories (42%); see Table 3. However, the CBF response varied widely and, in individual patients, could peak as late as 25 min after ACZ administration. In 68% of affected vascular territories, the maximum increase in CBF did not occur at 15 min post-ACZ injection. The mean difference between CVR at maximum CBF and 15 min post-ACZ injection in cases with missing peaks was 8 pp, with a range of 1–30 pp (*p* < 0.01). All individual CVRs and CBFs in affected and unaffected vascular territories are presented in Supplementary Tables 1, 2, respectively.

**Table 3:** Frequency distribution of maximum increase in CBF occurrence (%) for each individual vascular territory and all affected, unaffected, and unaffected contralateral vascular territories and all post-ACZ injection time points.

	**Affected**					**Unaffected**			**CL**
	**ACA_**left**_**	**ACA_**right**_**	**MCA_**left**_**	**MCA_**right**_**	**All**	**PCA_**left**_**	**PCA_**right**_**	**All**	**All**
5 min	29%	62%	37%	38%	42%	52%	65%	59%	61%
15 min	41%	24%	26%	38%	32%	24%	13%	18%	22%
25 min	29%	14%	37%	24%	26%	24%	22%	23%	17%

*ACA, anterior cerebral artery; CL, unaffected contralateral vascular territories; MCA, middle cerebral artery; PCA, posterior cerebral artery*.

**Figure 4:**
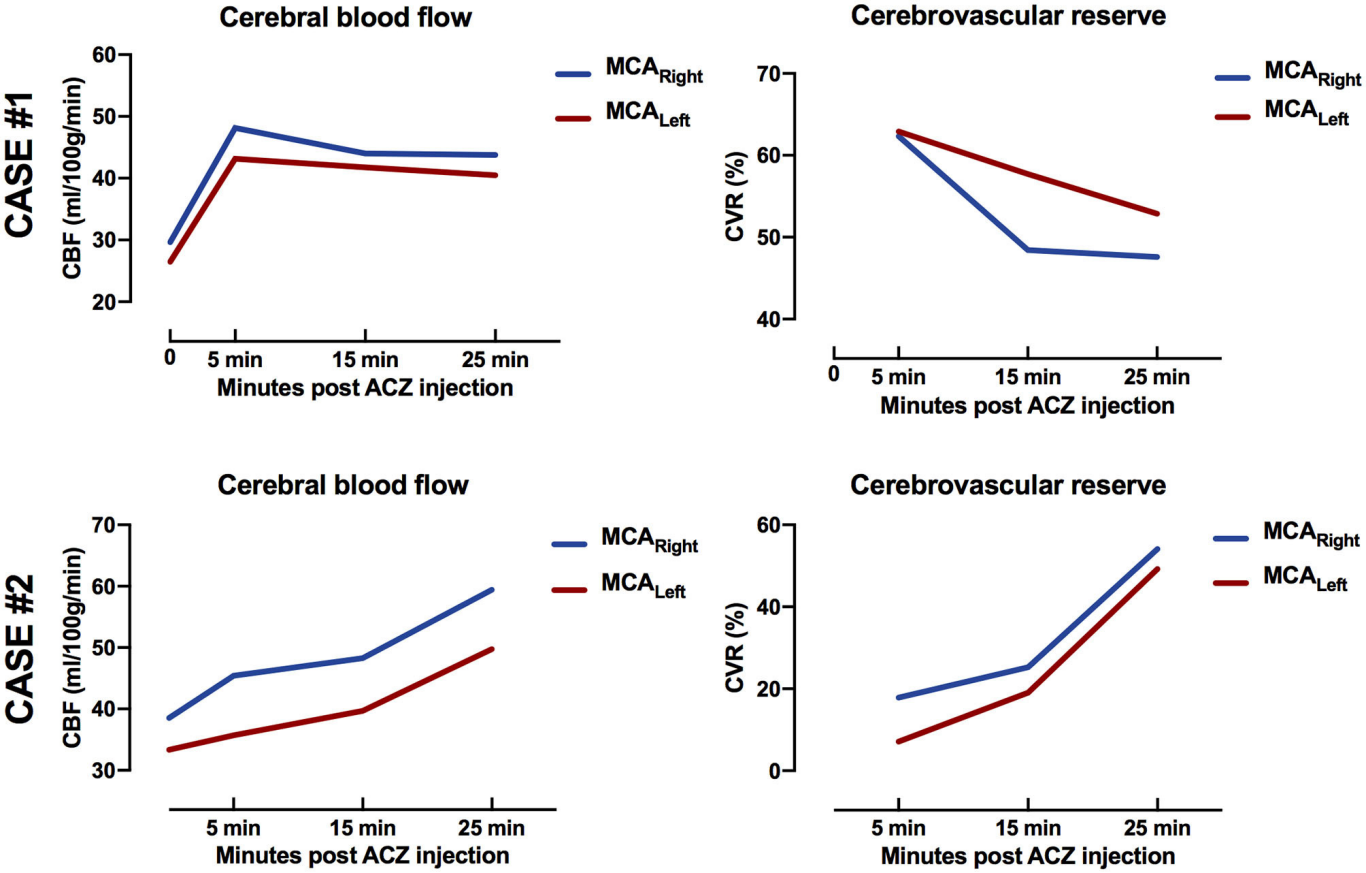
Case #1 (top row): Female, aged 51 years, with bilateral moyamoya disease [Suzuki grade III left internal carotid artery (ICA) and right ICA]. Magnetic resonance imaging was performed for preoperative evaluation. Temporal response of cerebral blood flow (CBF) and cerebrovascular reserve (CVR) peaked at 5 min post-acetazolamide injection. Case #2. (bottom row): Male, aged 14 years, with unilateral moyamoya disease (Suzuki grade II left ICA). Magnetic resonance imaging was performed after indirect revascularisation surgery of the left hemisphere. Temporal response of CBF and CVR increased for all time points post-acetazolamide injection. Notice difference between 15 min (left 19% and right 25%) and 25 min (left 49% and right 54%) post-acetazolamide injection.

CVR patterns are presented in two cases to illustrate the individuality of temporal response after ACZ injection (Figure 4). In case #1, both CBF and CVR reached a peak at 5 min post-ACZ injection and then decreased successively. In case #2, the CBF and CVR increased throughout all time-point post-ACZ injection. Notice the difference between 15 min (left 19% and right 25%) and 25 min (left 49% and right 54%) post-ACZ injection.

## Discussion

In this study, we investigated the temporal response of CBF and CVR during the ACZ challenge in vascular territories at baseline and 5, 15, and 25 min post-injection in patients with MMD using ASL. Major findings include that measurement of CBF at only a single time point after ACZ administration may underestimate CVR by up to 30 pp in individual cases and that unaffected vascular territories and unaffected contralateral vascular territories are more prone to the early maximum increase in CBF compared with affected vascular territories.

### Average Baseline and Post-acetazolamide Injection Cerebral Blood Flow and Cerebrovascular Reserve Capacity

CVR assessment using ACZ in patients with steno-occlusive diseases using ASL has been investigated by others. Yun et al., using pCASL with a post-label delay of 1,500 ms at 1.5 T, found an increase in CBF of 40 ml/100 g/min (baseline 35 ml/100 g/min) at 10 min post-ACZ injection in patients with MMD. Unfortunately, CVR was not calculated with absolute CBF values. Furthermore, Bokkers et al. (18), using flow territory-selective ASL acquisition in patients with carotid artery stenosis, reported an increase in CBF of 61 ml/100 g/min (baseline 61 ml/100 g/min, CVR = 36%) at 15 min post-ACZ injection. In contrast, Federau et al. (12) reported an increase in CBF of 65–67 ml/100 g/min (baseline 52–55 ml/100 g/min, CVR ranging from 24 to 36%) at 15 min post-ACZ injection in mildly/moderate to severely stenosed/occluded regions in patients with MMD using a multi-delay pCASL acquisition. Ni et al. (3) also used a multi-delay pCASL acquisition and reported an increase in CBF of 49 and 58 ml/100 g/min (baseline 49 ml/100 g/min, CVR = 21%) at 10 to 15 min post-ACZ injection in affected vascular territories in patients with MMD. In summary, other investigations using ASL have found similar baseline and post-ACZ injection CBF values (thus also CVR) in affected vascular or specific non-vascular regions (3, 12, 18, 30–32).

In addition to ASL, other imaging modalities such as single-photon emission computerized tomography, positron emission tomography (PET), and Xenon computed tomography have been used to assess CVR in patients with steno-occlusive diseases and healthy subjects, often with a single CBF measurement 5 to 20 min after ACZ injection. An average CVR of <40% is reported in affected vascular territories or hemispheres (3, 9, 12–19). In unaffected contralateral regions or hemispheres and unaffected vascular territories (healthy subjects), an average CVR of >40% (up to 70%) is commonly reported (3, 8, 9, 12, 18). CVR values from other investigations are presented in Supplementary Table 3.

Although reported group-averaged CVRs measured using different imaging modalities are similar in both affected and unaffected vascular territories/hemispheres in patients, a large dispersion is evident in the data (demonstrated as a coefficient of variation). Of note, a coefficient of variation of up to 217% has been reported in affected vascular territories. Furthermore, unaffected vascular territories in patients with unilateral disease and in healthy subjects reported CVR that shows a narrower dispersion compared with affected vascular territories; this is particularly well-demonstrated in studies including data from both patients and healthy subjects. A possible explanation for the large dispersion in group average CVR values may be related to an individual temporal response to ACZ injection.

### Temporal Response—When Is the Peak Expected

Few studies have assessed temporal CVR using multiple CBF measurements post-ACZ injection. Hartkamp et al. (7) concluded that the optimal timing for a single post-ACZ injection measurement for reliable assessment of CVR in patients with symptomatic ICA stenosis is between 15 and 20 min based on phase-contrast MRI measurements in the ICAs. An important limitation of that study was the lack of measured CBF at the regional cerebral level, which may explain differences compared with our results. In contrast, Okazawa et al. (10) found that increase in CBF was greater at 10 min post-ACZ injection compared with 20 min using ^15^O-water PET. Furthermore, Inoue et al. (8) reported that the maximum increase in CBF occurred at ~10 min post-ACZ injection in the ACAs, MCAs, and PCAs using ASL in healthy subjects. Hauge et al. (22) and Dahl et al. (21) reported similar results using transcranial Doppler in healthy subjects. Vaclavu et al. (11) studied the temporal response in healthy subjects and patients with sickle cell disease using ASL and reported that the maximum increase in CBF occurred at 10 to 15 min post-ACZ injection.

Kuwabara et al. (9) demonstrated, using ^15^O-water PET, that hemispheric CBF to ACZ was greater after 20 min than after 5 min in hemispheres affected by occlusion but not in unaffected hemispheres. The timing differences between affected compared with unaffected hemispheres found by Kuwabara et al. (9) are also supported by the findings of Hartkamp et al. (7). In comparison, our analysis of CVR distribution showed that unaffected and unaffected contralateral vascular territories are more prone to the early maximum increase in CBF than affected vascular territories (data shown in Table 3). This further adds to the possibility that the temporal response to ACZ injection may provide valuable clinical information (7, 9).

### Cerebrovascular Reserve Capacity Can Be Underestimated (Peak vs. 15 min)

We compared CVR derived from the actual maximum increase in CBF at 5, 15, or 25 min post-ACZ injection with the corresponding CVR derived from the commonly used time point of 15 min post-ACZ injection for each patient and vascular territory. We found that the maximum increase in CBF (and maximum CVR) did not occur at 15 min in 68% of all affected vascular territories. In these cases, there was a significant difference between CVR derived from an actual maximum increase in CBF when the peak was overlooked and CVR derived at 15 min post-ACZ injection. In these missing peak cases, CVR may be underestimated in the range of 1–30 pp (mean difference 8 pp) in individual patients.

### Implications of Underestimated Cerebrovascular Reserve

CVR is used in the individual clinical assessment of patients with MMD, but the criteria defining impaired CVR vary. Kuroda et al. (20) and Ogasawara et al. (33) both used a healthy control group to define an impaired CVR as a CBF response <14 and 19%, respectively. Acker et al. (34) defined it at 15% CVR (50% reduction from normal 30%). In a proposal for a new grading of MMD in adult patients (The Berlin Grading System), Czabanka et al. (35) defined severely impaired CVR as a reduced CBF response of more than 5% (equivalent to a negative CVR of 5% or more). This novel grading system was later validated by Teo et al. (36) in a larger patient cohort.

Caution is advised in respect to the lack of an explicit definition of normal and impaired CVR (6) and the variable temporal response in CBF post-ACZ injection discussed in this study with regard to the assessment of disease progression and indications for revascularisation surgery. Further studies using multiple CBF measurements post-ACZ injection are needed to assess the potential negative effects of variable individual CBF responses to ACZ and potential valuable clinical information.

### Limitations

The patients in the present study represent a heterogeneous population. Included patients are both adults and children with bilateral or unilateral MMD. Both pre- and postoperative examinations and examinations performed on the same patient are included in the analysis. This can obscure possible findings, and interpretation of the current results should be made with caution. We did not perform subgroup analysis to assess any temporal differences between pre- and postoperative examinations. Future larger patient cohorts are needed to address potential changes in temporal response after revascularisation procedures. Timing differences between anterior and posterior circulation can exist; this should be kept in mind when comparing values from affected and unaffected vascular territories in the current study.

Motion correction of the acquired ASL data could not be performed, as the CBF maps were calculated automatically by the scanner. The use of background suppression partly mitigates motion, and the control/label pairs were thoroughly visually inspected for possible motion. No exclusions were made due to excessive motion.

The reliability of the CBF measurements is especially vital during scan–rescan measurements aiming to detect different CVR at different time points after ACZ injection. In this sense, there can be uncertainty in distinguishing an actual temporal difference in CBF from a measurement error. Although we cannot rule out a reliability error as a confounder for temporal response in individual patients/examinations, there is no known data on reliability in CBF measurements after ACZ injection, and the presence of varying increases in CBF with time after ACZ injection is supported by other investigations as discussed earlier.

## Conclusion

There is a substantial variation in CVR between different time points after the ACZ challenge in MMD patients. Thus, there is a risk that the use of a single post-ACZ measurement time point overestimates disease progression, which could have large implications for decision-making regarding revascularisation surgery and the interpretation of the outcome thereof. Further studies with larger sample sizes using multiple CBF measurements post-ACZ injection in patients with MMD are encouraged.

## Data Availability Statement

The raw data supporting the conclusions of this article will be made available by the authors, without undue reservation.

## Ethics Statement

This retrospective study was done in accordance with the declaration of Helsinki and was approved by the Swedish Ethical Review Authority, and all included patients or legal guardian signed an informed consent.

## Author Contributions

MF performed data post-processing, statistical analysis, and drafting the manuscript. All authors contributed to the conception, design of the study, participated in the data collection, writing process, and approved the final version. All authors contributed to the article and approved the submitted version.

## Conflict of Interest

The authors declare that the research was conducted in the absence of any commercial or financial relationships that could be construed as a potential conflict of interest.
