# Supplementary material for: Variable Temporal Cerebral Blood Flow Response to Acetazolamide in Moyamoya Patients Measured Using Arterial Spin Labeling
Source: Front Neurol. 2021 Jun 8;12:615017. doi: 10.3389/fneur.2021.615017 (PMC8217767; doi:10.3389/fneur.2021.615017)
Supplement: Supplementary file 1 [file Data_Sheet_1.PDF]

Supplementary Material

Item	Page no.
Table 1. CBF for all timepoints and examinations	2
Table 2. CVR for all timepoints and examinations	4
Table 3. Compilation of results from other publications	6

Table 1. Average CBF in ml/100 g/min in all vascular territories at all time points (0 min – baseline, 5 min, 15 min and 25 min) for all subjects and examinations. Contralateral vascular territories in patients with unilateral moyamoya disease are annotated in bold.

#		ACA_{Left}				ACA_{Right}				MCA_{Left}				MCA_{Right}				PCA_{Left}				PCA_{Right}			
	Min	0	5	15	25	0	5	15	25	0	5	15	25	0	5	15	25	0	5	15	25	0	5	15	25
1	Post-op MRI	45	62	65	65	**56**	**79**	**82**	**80**	49	64	65	67	**58**	**75**	**75**	**79**	45	58	61	58	53	68	70	67
	Post-op MRI	39	65	63	59	**51**	**82**	**82**	**70**	43	71	67	60	**46**	**77**	**73**	**64**	32	59	59	48	39	69	69	55
	Post-op MRI	34	38	41	51	**44**	**49**	**53**	**67**	33	36	40	50	**39**	**45**	**48**	**59**	22	27	29	38	35	38	41	53
2	Post-op MRI	27	31	30	31	26	33	31	32	29	32	31	32	27	32	31	32	27	32	31	31	26	32	30	30
	Post-op MRI	27	29	31	29	26	33	33	32	28	31	32	31	28	32	33	32	-	-	-	-	-	-	-	-
	Post-op MRI	29	34	33	32	25	31	29	29	31	36	34	34	23	29	28	28	26	29	29	29	23	28	27	27
3	Post-op MRI	27	43	46	42	31	45	43	42	28	40	42	39	31	41	45	42	25	46	44	41	24	44	42	40
	Post-op MRI	31	45	40	39	34	46	44	44	29	41	38	38	30	42	37	39	26	43	37	37	26	41	36	34
	Post-op MRI	28	39	42	42	33	44	47	44	28	40	43	40	28	39	42	41	25	43	44	39	24	39	39	36
4	Pre-op MRI	**29**	**43**	**42**	**39**	27	37	38	35	**30**	**38**	**40**	**39**	28	34	35	35	21	32	31	29	21	31	30	29
	Post-op MRI	**25**	**40**	**37**	**37**	26	40	37	37	**29**	**42**	**40**	**40**	29	42	40	40	32	48	40	42	30	45	36	40
	Post-op MRI	**34**	**53**	**51**	**48**	33	48	47	44	**38**	**54**	**54**	**50**	35	45	47	43	27	44	43	37	26	42	41	36
5	Pre-op MRI	**37**	**58**	**58**	**54**	34	63	60	55	45	66	69	64	43	55	57	55	28	48	46	42	28	50	46	42
	Post-op MRI	**45**	**64**	**58**	**60**	34	64	57	56	53	70	67	66	50	64	63	60	38	55	50	48	25	47	45	42
6	Pre-op MRI	47	64	66	72	43	65	60	65	54	74	74	77	52	71	70	73	44	63	61	71	37	53	51	60
	Pre-op MRI	52	73	70	68	46	69	62	66	62	88	84	85	59	85	77	78	43	67	66	67	38	59	52	56
	Post-op MRI	52	74	75	78	47	70	70	73	60	87	88	93	60	88	89	92	47	70	68	73	39	62	61	63
	Pre-op MRI	**36**	**64**	**58**	**60**	34	63	60	55	**41**	**71**	**70**	**64**	42	75	74	67	-	-	-	-	28	50	46	42

7	Pre-op MRI	**34**	**68**	**61**	**59**	34	64	57	56	**41**	**75**	**69**	**65**	42	78	73	70	-	-	-	-	25	47	45	42
8	Pre-op MRI	27	45	41	42	28	46	43	42	28	46	43	42	30	48	44	44	24	39	37	35	25	40	37	36
9	Pre-op MRI	47	56	72	70	48	66	62	60	49	62	69	67	51	66	71	71	51	73	83	80	51	69	77	78
10	Post-op MRI	50	79	83	79	48	72	76	74	49	75	78	76	47	76	79	75	37	62	60	55	33	58	56	51
	Post-op MRI	34	52	54	52	32	48	50	51	35	51	53	54	35	52	54	54	31	49	48	46	22	36	36	36
11	Pre-op MRI	42	52	53	54	45	58	53	59	47	61	53	62	45	57	53	57	34	46	45	48	41	55	49	54

ACA, anterior cerebral artery; MCA, middle cerebral artery; MRI, magnetic resonance imaging; PCA, posterior cerebral artery

Table 2. Average CVR in percentage in affected and unaffected vascular regions at all time points (5 min, 15 min and 25 min) for all subjects and examinations. Contralateral vascular territories in patients with unilateral moyamoya disease are annotated in bold.

#		ACA_{Left}			ACA_{Right}			MCA_{Left}			MCA_{Right}			PCA_{Left}			PCA_{Right}		
	Min	5	15	25	5	15	25	5	15	25	5	15	25	5	15	25	5	15	25
1	Post-op MRI	37	44	42	**40**	**45**	**43**	29	32	37	**28**	**30**	**36**	28	35	28	29	34	28
	Post-op MRI	64	59	50	**60**	**61**	**37**	65	56	41	**68**	**59**	**38**	85	85	51	76	76	41
	Post-op MRI	10	21	50	**13**	**22**	**53**	7	19	49	**18**	**25**	**54**	22	33	75	10	17	53
2	Post-op MRI	13	11	13	26	18	23	10	7	10	17	16	19	19	14	15	25	19	18
	Post-op MRI	8	14	10	24	25	22	11	14	11	16	17	14	-	-	-	-	-	-
	Post-op MRI	14	11	10	23	16	14	14	10	8	25	22	21	12	13	13	22	19	18
3	Post-op MRI	58	67	54	46	40	37	42	50	41	29	42	34	82	77	63	81	71	64
	Post-op MRI	48	32	28	33	27	27	43	30	30	41	27	30	63	42	41	54	35	28
	Post-op MRI	37	49	49	35	43	33	41	52	40	41	51	48	77	80	60	66	66	55
4	Pre-op MRI	**50**	**44**	**36**	37	42	31	**28**	**34**	**30**	20	25	23	54	46	40	52	45	40
	Post-op MRI	**62**	**48**	**48**	53	44	42	**44**	**37**	**37**	43	36	37	48	23	29	48	18	31
	Post-op MRI	**55**	**47**	**40**	43	43	31	**41**	**43**	**32**	28	33	22	60	58	36	61	55	35
5	Pre-op MRI	**56**	**56**	**45**	44	45	37	47	53	43	29	33	28	71	66	52	59	45	35
	Post-op MRI	**42**	**29**	**34**	35	30	29	31	26	23	28	25	20	46	33	28	44	31	26
6	Pre-op MRI	37	41	53	49	39	50	37	38	43	37	35	42	43	40	61	46	40	64
	Pre-op MRI	39	34	30	48	34	42	41	35	36	45	31	33	57	54	59	56	39	48
	Post-op MRI	43	45	51	48	49	55	46	48	56	48	49	53	49	44	56	58	54	60
	Pre-op MRI	**86**	**78**	**66**	85	74	62	**75**	**71**	**57**	76	74	59	-	-	-	76	61	49

7	Pre-op MRI	**98**	**78**	**72**	89	69	65	**85**	**69**	**60**	85	74	65	-	-	-	86	78	68
8	Pre-op MRI	65	52	53	63	53	50	63	53	50	62	48	48	62	53	45	60	48	43
9	Pre-op MRI	19	52	48	39	30	27	26	40	36	28	39	38	43	63	56	36	52	53
10	Post-op MRI	57	64	57	51	59	54	53	58	54	62	69	60	68	64	49	75	68	55
	Post-op MRI	55	61	55	48	56	59	47	52	55	50	55	56	57	52	47	61	63	60
11	Pre-op MRI	25	27	28	31	18	33	31	14	33	24	17	25	36	32	42	34	19	31

ACA, anterior cerebral artery; MCA, middle cerebral artery; MRI, magnetic resonance imaging

Table 3. Compilation of cerebrovascular reserve (CVR) results from other investigations in patients with affected hemispheres, patients with unaffected hemispheres and normal subjects. Mean CVR (%) and standard deviation (SD) are included when available. Standard error was re-calculated to standard deviation by multiplying with the square root of the number of subjects. Coefficient of variation (CoV) was calculated by dividing SD with mean CVR. Imaging methods include positron emission tomography (PET), arterial spin labelling (ASL) with different post-label delays (PLD), single-photon emission computed tomography (SPECT). Measurement time point after acetazolamide administration is included in min. Type I is classified as normal CBF and CVR, type II normal CBF and reduced CVR and type III reduced CBF and CVR. Values from the current study are annotated in bold.

	Study	Mean	SD	CoV	Method	Comment
Patients Affected Hemispheres	Federau 2017	36	52	144%	ASL 15 min	Moderate Stenosis
	Federau 2017	24	52	217%	ASL 15 min	Severe Stenosis
	Hu 2017	25	30	121%	ASL 10–15 min	
	Ni 2017	21	38	181%	ASL 10–15 min	
	Noguchi 2015	31	40	131%	SPECT 20 min	
	Noguchi 2011	36	44	123%	SPECT 20 min	
	Bokkers 2010	36	14	40%	ASL 15 min	
	Uchihashi 2010	32	24	76%	SPECT 10 min	
	Uchihashi 2010	32	31	98%	ASL 10 min	
	Kuroda 2001	-4	5	89%	SPECT 15 min	Type III
	Kuroda 2001	5	2	44%	SPECT 15 min	Type II
	Kuroda 2001	19	4	21%	SPECT 15 min	Type I
	Detre 1999	35	-	-	ASL 15 min	
	Kuwabara 1995	31	23	75%	PET 20 min	
	Kuwabara 1995	24	22	95%	PET 5 min	
	**Current study**	**39**	**17**	**43%**	**ASL 5, 15, 25 min**	
Patients Unaffected Hemisphere	Federau 2017	45	45	100%	ASL 15 min	
	Ni 2017	41	26	63%	ASL 10-15 min	
	Bokkers 2010	57	19	33%	ASL 15 min	
	Bokkers 2010	45	17	38%	ASL 15 min	
	Uchihashi 2010	36	23	66%	SPECT 10 min	
	Uchihashi 2010	38	28	73%	ASL 10 min	
	Detre 1999	40	-	-	ASL 15 min	
	Kuwabara 1995	40	22	55%	PET 20 min	
	Kuwabara 1995	38	20	52%	PET 5 min	
	**Current study**	**49**	**18**	**38%**	**ASL 5, 15, 25 min**	
	Kuttner 2021	31	29	94%	PET 15-30 min	
	Vaclavu 2020	67	20	30%	ASL 10 min	
	Zhao 2020	63	13	21%	ASL 10 min	
	Vaclavu 2019	70	32	47%	ASL 10 min	

Normal Subjects	Inoue 2013	31	8	25%	ASL Dynamic	PLD 1525 ms
	Inoue 2013	46	10	22%	ASL Dynamic	PLD 2525 ms
	Bokkers 2010	70	22	31%	ASL 15 min	Basilar Artery
	Bokkers 2010	48	14	29%	ASL 15 min	ICA
	Kuroda 2006	30	8	27%	SPECT 15 min	
	Endo 2006	35	10	29%	SPECT	
	Yen 2002	38	24	65%	ASL 12 min	
	Ogasawara 2001	28	4	16%	PET 10 min	
	Kuroda 2001	20	5	26%	SPECT 15 min	
	Okazawa 2001	34	-	-	SPECT	

ACA, anterior cerebral artery; ASL, arterial spin labelling; CoV, coefficient of variation; ICA, internal carotid artery; MCA, middle cerebral artery; PCA, posterior cerebral artery; PET, position emission tomography; PLD, post-label delay; SD, standard deviation; SPECT, single-photon emission tomography;
